# CMOS Point-of-Care Diagnostics Technologies: Recent Advances and Future Prospects

**DOI:** 10.3390/mi15111320

**Published:** 2024-10-29

**Authors:** Tania Moeinfard, Ebrahim Ghafar-Zadeh, Sebastian Magierowski

**Affiliations:** 1 Department of Electrical Engineering and Computer Science, Lassonde School of Engineering, York University, Toronto, ON M3J 1P3, Canada; tmd12@yorku.ca (T.M.); magiero@yorku.ca (S.M.); 2 Biologically Inspired Sensors and Actuators (BioSA) Laboratory, York University, Toronto, ON M3J 1P3, Canada; 3 Electronic Machine Intelligence Lab, York University, Toronto, ON M3J 1P3, Canada

**Keywords:** point-of-care (PoC), CMOS technology, biomedical integrated circuits (ICs), bioimpedance spectroscopy, SARS-CoV-2 diagnostics, glucose monitoring, neural recording, medical diagnostics, portable devices, real-time monitoring

## Abstract

This review provides a comprehensive overview of point-of-care (PoC) devices across several key diagnostic applications, including blood analysis, infectious disease detection, neural interfaces, and commercialized integrated circuits (ICs). In the blood analysis section, the focus is on biomarkers such as glucose, dopamine, and aptamers, and their respective detection techniques. The infectious disease section explores PoC technologies for detecting pathogens, RNA, and DNA, highlighting innovations in molecular diagnostics. The neural interface section reviews advancements in neural recording and stimulation for therapeutic applications. Finally, a survey of commercialized ICs from companies such as Abbott and Medtronic is presented, showcasing existing PoC devices already in widespread clinical use. This review emphasizes the role of complementary metal-oxide-semiconductor (CMOS) technology in enabling compact, efficient diagnostic systems and offers insights into the current and future landscape of PoC devices.

## 1. Introduction

Point-of-care (PoC) diagnostics are becoming increasingly vital in addressing global healthcare challenges, particularly in rural and underserved areas where access to healthcare is limited [1,2]. The COVID-19 pandemic, which began in December 2019, brought unprecedented strain on healthcare systems worldwide, and highlighted the need for rapid diagnostic tools [3,4,5]. As with previous pandemics, such as the 1918 influenza [6,7], HIV [8,9], and SARS [10,11], the need for immediate diagnostic capabilities became clear. PoC diagnostics can provide real-time, on-site testing that is crucial for containing outbreaks and initiating treatment early. This is particularly important in low-resource settings where traditional laboratory infrastructure is not always available [12].

PoC diagnostics play a critical role beyond pandemic response. These devices allow for timely diagnosis and management of chronic diseases [12,13], and they hold the potential to revolutionize healthcare by reducing the burden on hospitals and clinics, providing immediate results, and enabling remote monitoring. In rural areas, PoC devices can bridge the gap in healthcare access, improving patient outcomes by facilitating early detection and intervention [1]. With the global rise in chronic diseases and the continued threat of infectious outbreaks, PoC diagnostics are poised to play a crucial role in the future of healthcare.

An essential enabler of these point-of-care (PoC) devices is CMOS technology, which plays a pivotal role in the development of compact, power-efficient, and cost-effective diagnostic tools. CMOS technology is known for its scalability, allowing for the seamless integration of various functionalities—such as sensing, signal processing, and data communication—onto a single chip. This high level of integration makes CMOS an ideal choice for PoC applications, where portability, efficiency, and cost are critical considerations [14].

One of the key advantages of CMOS is its ability to support multi-functional systems on a chip (SoC), enabling researchers to develop devices capable of performing complex diagnostic tests in a single, portable format unlike traditional testing, which requires different equipment, is bulky, expensive, and not at the point of care [15]. The tests that CMOS PoC enable can range from blood analysis and neural recording to the detection of pathogens and biomarkers associated with various diseases. For instance, CMOS technology allows the integration of multiple sensor types alongside essential analog and digital circuits, reducing the need for bulky external equipment and making these devices more user-friendly and accessible in resource-limited environments [16,17,18,19].

In PoC devices, the miniaturization and low-power consumption of CMOS circuits make them particularly suited for battery-powered or energy-harvesting applications, which are often necessary in remote or mobile health setups [20,21]. Furthermore, CMOS fabrication techniques are well-established and cost-effective, leveraging the existing semiconductor manufacturing infrastructure to produce devices at scale with relatively low costs [18,22]. This mass manufacturability is essential for the widespread adoption of PoC devices, ensuring that they remain affordable for large-scale deployment, particularly in low-resource settings [23,24].

By leveraging CMOS, researchers can also incorporate advanced signal processing capabilities directly into the device, enhancing the accuracy and sensitivity of diagnostic tests. For example, PoC devices for blood analysis can measure specific biomarkers like glucose or proteins with high precision, while neural recording devices can capture intricate brain activity patterns in real time. Similarly, CMOS-enabled pathogen detection systems can rapidly identify infectious agents by integrating biosensors with on-chip signal amplification and data processing [20,25].

**Figure 1:**
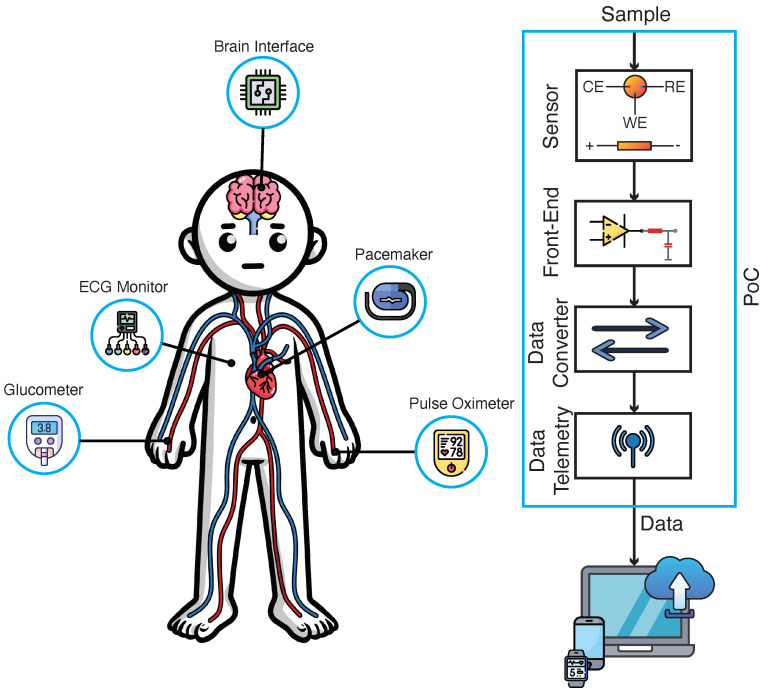
Various point-of-care devices, illustrating the integration of sensors for different health monitoring applications. The point-of-care block diagram on the right shows the main blocks and communication with our devices for real-time analysis and remote healthcare management.

Figure 1 illustrates several examples of PoC devices and a block diagram outlining their primary components. The block diagram typically includes sensing elements, analog front-ends for signal conditioning, data converters (e.g., ADCs), signal processors, power management units, and wireless communication modules. Each of these components is vital to the overall function of the PoC device, working together to provide real-time diagnostic capabilities in a compact and user-friendly form factor [14].

In this review, we explore the integration of CMOS technology into PoC devices, addressing the critical circuit design challenges across various diagnostic applications such as blood analysis, neural recording, and therapeutic drug monitoring. While existing reviews, such as those by Kubina et al. [26], Li et al. [27], and Li et al. [28], have largely focused on specific applications or diagnostic technologies, they often neglect the engineering complexities of circuit implementation that are essential for creating efficient, low-power PoC systems. These works emphasize individual diagnostic tools or biosensor development without delving into the integration of sensing, signal processing, and data communication into a cohesive, miniaturized platform. In contrast, our review takes a more comprehensive approach by highlighting the role of CMOS technology in enabling these compact, high-performance devices. We address not only the functionality of PoC devices but also the critical circuit-level advancements that make them scalable and effective in real-world medical scenarios. By focusing on the integration of sensors with CMOS circuits and addressing challenges such as power consumption, signal fidelity, and on-chip data telemetry, this review provides a detailed technical perspective that fills a gap in the current literature and offers valuable insights for the future development of PoC systems.

This paper is organized as follows. Section 2 focuses on blood analysis. In Section 3, we examine infectious disease detection. Section 4 delves into the development of PoC devices for neural interface. In Section 5, we highlight commercialized PoC devices that have transitioned from research prototypes to clinically used products. In Section 6, the future direction of PoC devices is discussed and finally, in Section 7, we have a brief conclusion.

## 2. Blood Analysis

Blood analysis is a cornerstone of medical diagnostics, providing essential insights into a patient’s health by detecting a wide range of biomarkers [29,30,31,32]. It plays a vital role in disease management and prevention, from monitoring glucose levels in diabetic patients to tracking dopamine concentrations for neurological studies. In this section, we will explore the detection of key analytes with a focus on the latest advancements in these areas. Additionally, we will introduce other devices that utilize whole blood for various diagnostic applications, demonstrating the diverse technologies available in blood analysis today.

**Figure 2:**
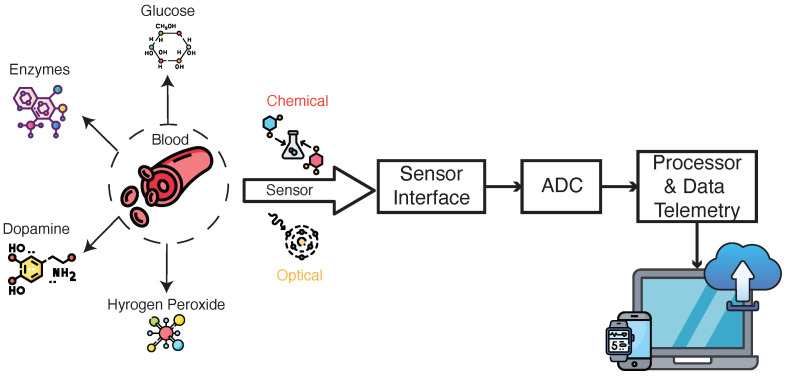
Blood analysis principle.

The overview of point-of-care (PoC) devices for blood analysis is depicted in Figure 2. This figure highlights two primary types of sensors: biochemical and optical sensors. These sensors detect key analytes present in the blood. The detected signals from the sensors are processed through an analog interface, where they are converted into digital signals by an analog-to-digital converter (ADC). The processed data are then transmitted to a processor for further analysis and communication, ensuring that the collected information can be wirelessly transmitted for real-time monitoring and diagnosis. The integration of these sensors and telemetry components is crucial for effective and efficient blood analysis in PoC settings.

### 2.1. Target Biomarkers

In this section, some of the most widely used biomarkers are introduced and their application is discussed.

#### 2.1.1. Dopamine (DA)

DA is a critical neurotransmitter involved in regulating cognitive functions, motor control, and emotional responses. Abnormal dopamine levels are associated with neurological disorders such as Parkinson’s disease, schizophrenia, and depression. Therefore, the sensitive and reliable real-time detection of dopamine is crucial for the early diagnosis, monitoring, and treatment of these disorders. Point-of-care (PoC) systems capable of monitoring dopamine levels can provide personalized medical care, with the potential to significantly improve patient outcomes [33,34,35,36]. A range of electrochemical sensors has emerged as the primary technique for dopamine detection [33,34,37].

#### 2.1.2. Aptamers

Aptamers are short, single-stranded DNA or RNA molecules that bind to specific targets, such as proteins or small molecules, with high affinity and specificity. Their ability to form unique three-dimensional structures allows them to mimic antibody binding properties, making them valuable for therapeutic drug monitoring (TDM) and biosensing applications. Unlike antibodies, aptamers are synthetically produced, offering advantages like lower costs, easier modification, and enhanced stability. In TDM, aptamers detect and quantify drugs in the bloodstream, enabling real-time analysis of drug efficacy and dosage adjustments [38,39]. Continuous real-time monitoring is crucial for managing drugs with narrow therapeutic windows, where small deviations can lead to toxicity or therapeutic failure. Point-of-care (PoC) devices using electrochemical sensors fill this gap by providing accurate, continuous drug monitoring in bodily fluids [40,41].

#### 2.1.3. Glucose

Glucose is a simple sugar and the primary source of energy for the body’s cells. It is obtained from carbohydrates in the diet and is transported through the bloodstream to be used by cells for metabolism. The regulation of glucose levels in the blood is critical for maintaining normal bodily functions. Insulin, a hormone produced by the pancreas, helps cells absorb glucose from the blood, and abnormalities in this process can lead to conditions like diabetes. Monitoring glucose levels is essential for managing diabetes, where blood glucose can be too high (hyperglycemia) or too low (hypoglycemia) [42]. Recent advancements in wearable glucose sensors have introduced the potential for more accessible, pain-free monitoring methods [43,44,45].

### 2.2. Applied Detection Techniques

#### 2.2.1. Dopamine Detection

A range of electrochemical sensors has emerged as the primary technique for dopamine detection, with different methodologies employed to enhance sensitivity, flexibility, and integration with CMOS technology [33,34,37]. Graphene-based sensors, in particular, have been popular for their high surface area and conductivity. For instance, the flexible electrochemical sensor developed by [34] employs a hybrid architecture consisting of negatively charged graphene (GP) and electrostatically neutral gold nanoparticles (AuNPs), designed to significantly improve dopamine (DA) detection sensitivity. By leveraging the synergy between graphene’s π-π stacking interactions and the enhanced electron transfer kinetics provided by AuNPs, the sensor achieves nanomolar-level detection limits (LOD) for dopamine (10 nM), which is crucial for detecting DA concentrations in physiological conditions such as the extracellular fluid of the caudate nucleus in neurodegenerative disorders like Parkinson’s disease. The graphene-gold nanoparticle interface facilitates enhanced mass transfer and separation of voltammetric peaks due to a combination of electrostatic interactions and diffusion gradients, as illustrated by the distinct current peaks achieved in solutions coexisting with ascorbic acid (AA), DA, and uric acid (UA). Despite these advances in sensitivity and selectivity, the sensor architecture remains limited by its reliance on external circuitry for signal processing, as it lacks an integrated communication module for data telemetry. This omission poses challenges for its deployment in real-time, wearable biomedical systems, where fully integrated system-on-chip (SoC) designs are critical. Additionally, although the sensor is theoretically capable of whole blood detection, experimental verification of its performance in such complex biological matrices remains unconfirmed. The lack of validation in these environments raises concerns about biofouling, interference from complex biomolecules, and stability in long-term applications. As such, further optimization and integration of on-chip processing units are required to transition this sensor from a laboratory setting to practical clinical diagnostic [34]. CMOS-based approaches offer superior integration capabilities, particularly in the combination of signal processing, data telemetry, and low-noise amplification circuits, when compared to traditional graphene-gold nanoparticle sensors. The open-gate ion-sensitive field-effect transistor (ISFET) developed by [37] achieves femtomolar sensitivity for dopamine detection, leveraging a post-CMOS fabrication process that removes the polysilicon gate to expose the gate oxide for surface functionalization. The sensor utilizes 4-carboxyphenylboronic acid (CPBA) for selective dopamine binding, resulting in a detectable shift in threshold voltage. On-chip signal amplification is achieved through a self-oscillating readout circuit, enabling real-time detection with high precision and minimal external noise interference. In addition to its remarkable sensitivity, the ISFET design integrates fully with CMOS readout circuitry, allowing for real-time data acquisition and processing across multiple sensors simultaneously. This system achieves stable detection across a dynamic range, making it particularly advantageous for clinical applications where precision and low-noise measurements are paramount. Furthermore, the open-gate architecture, combined with advanced post-CMOS etching techniques, enables the sensor to operate at sensitivity levels comparable to or exceeding those of nanowire FETs, without the complexities and costs associated with nanowire fabrication. Another approach, the graphene ink-based electrochemical sensor presented by [46], integrates a low-cost, portable potentiostat that can be controlled wirelessly via a smartphone using Wi-Fi communication. The sensor utilizes a flexible polyimide substrate with spin-coated graphene ink, demonstrating dopamine detection down to 100 nM in phosphate-buffered saline (PBS). While the system benefits from remote data transfer capabilities, it remains less sophisticated compared to fully integrated CMOS designs due to its reliance on discrete components and breadboard prototyping. Comparing these techniques, the CMOS-based ISFET system [37] stands out for its complete integration, making it the most advanced for clinical real-time dopamine detection. The hybrid CMOS-graphene sensor [33] shows potential for PoC applications with its programmable noise reduction and fast response but requires further validation with whole blood. The graphene-gold nanoparticle sensor [34], while flexible and suitable for wearable applications, lacks the precision and communication capabilities needed for clinical use. Finally, Ref. [47] presents a UWB-based system for monitoring dopamine in small animals, emphasizing energy efficiency and low-power operation, though it is not designed for whole blood analysis. Overall, CMOS-based systems, particularly ISFETs, offer the most robust solution for clinical diagnostics, while hybrid and graphene-based sensors provide flexibility for wearable or PoC applications.

#### 2.2.2. Therapeutic Drug Monitoring

Aptamers are widely used to detect drug molecules by binding specifically to target drugs, generating a redox current (i-redox) that is measured using three-terminal electrochemical sensors [40,41]. The primary challenge in this application is accurately capturing and processing the i-redox current. Current conveyor circuits, designed to handle currents as small as a few nanoamps like i-redox, are utilized to minimize signal distortion. The captured signal is then processed by a dual-slope analog-to-digital converter (ADC), which integrates the signal over time to reduce noise and improve measurement accuracy. This integration is critical for biochemical sensing, where signals are small and noise is a major concern.

In [41], a sample-and-hold (S/H) technique is employed to reduce power consumption, making the sensor system more energy-efficient for continuous monitoring. This system integrates the front-end, amplifier, current conveyor, and dual-slope ADC on-chip, enabling efficient on-chip signal processing. In contrast, the system in [40] incorporates wireless power and data telemetry, making it the first continuous drug monitoring system with such capabilities. It also integrates the current conveyor, amplifier, and dual-slope ADC on-chip, but goes further by including telemetry, making it ideal for implantable applications requiring long-term monitoring without physical connections [40].

Both systems represent significant advancements in continuous therapeutic drug monitoring. The Sample-and-Hold CMOS Electrochemical Sensor [41] focuses on power efficiency, making it suitable for portable, low-power applications. Meanwhile, the Aptamer-Based Implantable Sensor [40] offers a more comprehensive solution with wireless power and telemetry, positioning it as a superior option for implantable, long-term monitoring applications. While both systems excel in continuous monitoring, the second system provides an edge in full-system integration and telemetry capabilities.

#### 2.2.3. Glucose Monitoring

Recent developments have spurred the evolution of glucose monitoring technologies into two primary categories: biochemical sensors and optical-based sensors [43,44,45].

**Figure 3:**
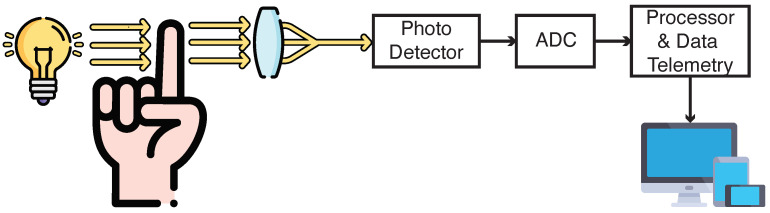
A schematic representation of an optical sensing system for health monitoring. Light is transmitted through the finger and detected by a sensor, followed by signal processing through a front-end circuit and analog-to-digital converter (ADC). The processed data are transmitted via a processor and data telemetry to external devices, such as a computer or smartwatch, for real-time monitoring and analysis.

- Biochemical Sensors
  Biochemical sensors, particularly electrochemical glucose sensors, form the backbone of many continuous glucose monitoring systems. These systems utilize the enzymatic reaction of glucose oxidase, where glucose is oxidized and produces hydrogen peroxide as a byproduct. This reaction generates a current proportional to the glucose concentration, which is then detected by the system.
  A prominent example is the Wireless Implantable Microsystem for continuous blood glucose monitoring [45]. This system integrates a microfabricated glucose biosensor flip-chip mounted onto a transponder chip. The glucose oxidase catalyzes a reaction that generates a measurable current within a range of 1 nA to 1 µA, maintaining accuracy with less than 0.3% non-linearity. The current is processed using a current-to-frequency converter, which provides power-efficient signal processing, essential for continuous monitoring. Data are transmitted wirelessly through load shift keying (LSK) and sent to an external reader via inductive coupling at 13.56 MHz, demonstrating effective performance in vitro. In [48], the same biochemical principle is employed. This design also detects hydrogen peroxide produced by the glucose oxidase reaction. It also uses a current-to-frequency converter and manages to reduce power consumption to just 4 µW, making it more suitable for long-term implantable applications. In addition to wireless power and data telemetry, this system enhances real-time glucose monitoring for extended periods. Both systems offer high accuracy by directly measuring the electrochemical reactions of glucose in the body, but they rely on invasive techniques that involve blood or interstitial fluid contact. When comparing these biochemical sensors, it is clear that both focus on precision, leveraging current-to-frequency converters to reduce power consumption while still delivering highly accurate glucose concentration measurements. Ref. [45] relies heavily on efficient signal processing to maintain power efficiency while ensuring accurate continuous monitoring, whereas [48] extends this approach, emphasizing ultra-low-power operation for prolonged use in implantable devices. Although both systems are effective for real-time glucose monitoring, their invasive nature limits patient comfort, making them less favorable for those seeking pain-free alternatives.
- Optical Sensors
  Optical glucose monitoring systems offer non-invasive methods for measuring glucose concentrations by utilizing light-based techniques to detect glucose in blood or interstitial fluid. Figure 3 shows this technique of sensing. These systems provide significant advantages in terms of patient comfort but face challenges with accuracy, particularly in managing noise and signal interference. For example, in one study [49], near-infrared (NIR) transmission spectroscopy was used to detect glucose levels, where NIR light is transmitted through the skin, and glucose molecules in the blood absorb specific wavelengths. The light signal is captured by a photodetector and processed using an analog front-end circuit for amplification and filtering, with data transmitted wirelessly via Bluetooth for real-time monitoring on smartphones. Although promising for non-invasive applications, further calibration is required to improve accuracy and ensure reliable glucose readings.

Another approach explored in [44] involves using photoplethysmography (PPG) to capture pulse wave signals through a smartphone camera. These signals are then processed with machine-learning algorithms, specifically support vector machines (SVMs), to estimate glucose levels. This provides a low-cost, non-invasive solution, eliminating the need for specialized hardware. However, reliance on machine learning introduces variability in accuracy, as the system remains susceptible to noise and demands robust algorithms for reliable performance. This is a significant difference when compared to biochemical sensors that provide direct measurements.

Further non-invasive methods discussed in the review by Siddiqui et al. [43] include wearable sensors using microneedles or skin patches to access interstitial fluid, where enzyme-based electrochemical sensors measure glucose levels. These sensors transmit the processed signals wirelessly through Bluetooth, allowing continuous monitoring without requiring blood samples. While more comfortable for patients, these systems are still being refined for clinical accuracy, and improvements are necessary to match the precision of invasive biochemical sensors. Biochemical and optical sensors each present a trade-off between accuracy and patient comfort. Biochemical sensors, such as the Wireless Implantable Microsystem [45] and the 4-Microwatt ADPLL-Based Biosensor [48], achieve high accuracy by directly measuring glucose through enzymatic reactions. These sensors are particularly beneficial for patients in critical conditions requiring real-time glucose monitoring. However, they are invasive, involving direct contact with blood or interstitial fluid, which can cause discomfort during long-term use.

In contrast, optical sensors, like those in [44,49], offer a more comfortable, non-invasive option but with lower accuracy. Light-based methods are prone to noise and interference, necessitating advanced signal processing and machine learning to ensure reliability. While these sensors show potential for continuous, pain-free glucose monitoring, their accuracy lags behind that of biochemical systems. However, they are ideal for wearable applications where ease of use and non-invasiveness are priorities.

In conclusion, biochemical glucose monitoring systems provide superior accuracy, making them ideal for critical care, while optical systems offer greater comfort and convenience, making them better suited for daily, non-invasive use. The choice between these technologies depends on the patient’s need for precision versus comfort in managing glucose levels.

#### 2.2.4. Other Techniques

In this section, we explore various devices designed for blood analysis, each targeting different substances for detection and measurement.

The first example is a device for assessing hemostasis, where a microfluidic dielectric sensor called ClotChip measures blood clotting properties through dielectric spectroscopy. ClotChip evaluates the electrical properties of blood as it clots, providing real-time feedback on clot formation. This innovation is especially important in managing patients at risk of thrombosis or those undergoing anticoagulant therapy. With ClotChip’s rapid and accurate clotting assessment, healthcare providers can monitor blood clotting disorders at the point of care, making it a valuable tool in both clinical and emergency settings. By integrating a microfluidic design with dielectric spectroscopy, ClotChip minimizes the need for large volumes of blood, ensuring low power consumption while providing high-precision data on clot formation [50].

Another critical advancement is the smartphone-based continuous-wave Doppler system, which provides real-time blood flow measurement. This system uses a Doppler sensor interfaced with a smartphone for data acquisition, analysis, and display. Continuous-wave Doppler technology enables accurate assessment of blood circulation, making it particularly useful in detecting blood flow abnormalities. The system’s portability and capability for clinical and remote use demonstrate how mobile technology can be integrated into healthcare, making blood flow monitoring accessible and easy to use in various environments [51].

One particularly notable PoC device is the BioMote, an injectable sensor capable of real-time alcohol and pH level monitoring. This system operates on an enzymatic reaction in which alcohol oxidase catalyzes the conversion of ethanol to hydrogen peroxide, which is then measured electrochemically to determine alcohol concentration. Notably, BioMote integrates multi-parameter sensing, allowing it to monitor pH changes alongside ethanol detection. This functionality is particularly important, as alcohol consumption can alter the body’s pH balance. The BioMote system employs a current-to-frequency converter to minimize power consumption, making it suitable for extended operation within the body. Its data telemetry uses backscattering technology, enabling wireless data transmission to an external reader. This power-efficient design and wireless communication make BioMote an advanced solution for continuous alcohol monitoring. In vitro and in vivo tests demonstrate the device’s effectiveness in real-world physiological conditions, making it a valuable tool for clinical applications and long-term alcohol monitoring programs [52].

A separate innovation, the OG-JFET sensor, demonstrates the potential of CMOS-based PoC devices for measuring critical biological parameters like pH and ion concentration. This ion-sensitive field-effect transistor (ISFET) sensor was developed using standard microfabrication processes, making it scalable and cost-effective for mass production. The sensor’s ability to measure blood pH accurately has broad applications in clinical diagnostics, particularly in monitoring the body’s acid-base balance. Deviations in blood pH levels are indicative of underlying health issues, such as metabolic or respiratory disorders, making the OG-JFET a critical tool for PoC devices. Its stability and sensitivity in detecting pH and ion concentrations add considerable value to PoC diagnostics [53].

Table 1 shows a summary of some of the state-of-the-art CMOS chips developed for blood analysis.

### 2.3. Circuit Discussions

**Figure 4:**
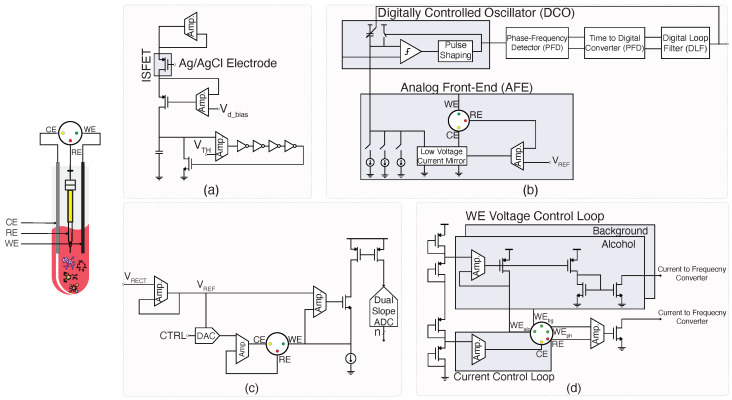
(**a**) OG-ISFET transistors for ultrasensitive dopamine detection [37]. (**b**) ADPLL-based implantable amperometric biosensor interface [48]. (**c**) Traditional interface of biochemical sensor for drug-monitoring applications [41]. (**d**) Injectable BioMote for continuous alcohol interface [52].

The circuits shown in Figure 4 demonstrate a variety of electrochemical sensing and signal processing architectures tailored for different biomedical applications, such as dopamine detection, drug monitoring, and alcohol sensing.

#### 2.3.1. OG-JFET Interface

The circuit in panel (a) features an Open-Gate Ion-Sensitive Field-Effect Transistor (OG-ISFET) that is highly sensitive to dopamine concentrations. In this configuration, the ISFET is paired with an Ag/AgCl reference electrode to provide stable electrochemical measurements [37]. The ISFET responds to changes in ion concentrations related to dopamine binding, generating a potential difference at the gate. This signal is then amplified and processed through a series of amplifiers, with a threshold voltage ($V_{T}H$) used for precise detection. This setup is ideal for low-concentration analyte detection due to its high sensitivity and real-time response capability, critical for continuous dopamine monitoring in clinical settings.

#### 2.3.2. DCO-Based Biochemical Sensor Interface

Panel (b) showcases a digitally controlled oscillator (DCO) used in conjunction with an all-digital phase-locked loop (ADPLL) to achieve precise control of the biosensor interface for drug monitoring applications [48]. The ADPLL serves as a key component for controlling the phase and frequency of the oscillator, which in turn drives the biosensor. The analog front-end (AFE) processes the input signals from the working (WE), reference (RE), and counter (CE) electrodes, converting them to voltage signals that are then shaped for subsequent digital conversion. The AFE, along with the ADPLL, ensures that the biosensor operates with minimal phase noise and high-frequency stability, making this system suitable for implantable biosensors where power and noise optimization are crucial.

#### 2.3.3. Traditional Interface for Biochemical Sensing

In panel (c), the traditional analog interface for biochemical sensors is illustrated used in [40,41]. The system comprises a low-voltage current mirror that interfaces with the electrochemical cell through the working, reference, and counter electrodes. A digital-to-analog converter (DAC) is used to control the amplitude of the applied voltage, while the output signal is amplified and converted using a dual-slope ADC. This setup enables high-precision detection of drug concentrations in the blood by continuously monitoring redox reactions. This traditional interface, though reliable, is relatively power-hungry, making it less suitable for long-term, low-power applications like wearable sensors. Panel (d) illustrates another biochemical sensor interface [54]. This system which is very similar to panel (c) leverages a working electrode (WE) voltage control loop to isolate the background noise from the actual signal. A current-to-frequency converter is used to digitize the output, allowing for precise measurement of blood alcohol content in real time. This system is designed for implantation, making the low-power current control loop essential for ensuring long-term operation without frequent battery replacements. The modular nature of the BioMote design also allows for integration with wireless telemetry systems, making it suitable for continuous monitoring in point-of-care applications.

## 3. Infectious Disease Detection

The application of PoC diagnostics for infectious diseases is of paramount importance, particularly in addressing global health challenges like the COVID-19 pandemic. PoC devices enable rapid, on-site testing, which is crucial for timely diagnosis, treatment, and containment of infectious diseases. For example, in the case of Hepatitis C, PoC testing has significantly improved the detection and management of the disease, reducing transmission rates and enabling quicker intervention [55,56].

**Figure 5:**
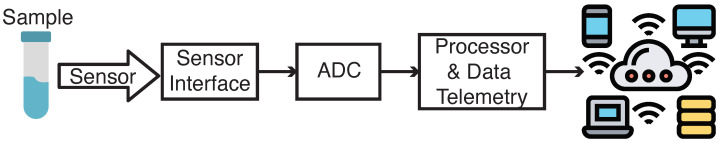
Block diagram of infectious disease detection PoC devices.

Moreover, PoC diagnostics are essential in community settings, where access to central laboratory facilities may be limited, especially in resource-constrained regions. PoC devices for infectious diseases have been instrumental in improving healthcare delivery in such settings, as seen with community-based programs targeting Hepatitis C and other viral infections [57]. The rapid detection and cost-effectiveness of PoC systems not only help in early disease management but also in large-scale screening efforts that are vital for epidemic and pandemic preparedness. The overview of PoCs for infectious diseases is shown in Figure 5. As shown, the sensor converts chemical reactions to electrical signals then they are amplified, digitized, and sent to electronic devices for further processing.

CMOS technology plays a significant role in enhancing these PoC devices by providing the benefits of integration, miniaturization, and cost-effectiveness. The ability to integrate multiple sensing, processing, and communication functions on a single chip makes CMOS-based PoC devices highly suitable for large-scale deployment, particularly in low-resource settings where traditional laboratory-based testing may not be feasible. By offering advanced diagnostic capabilities at a lower cost, CMOS-enabled PoC diagnostics can help mitigate the spread of infectious diseases and contribute to global health initiatives aimed at improving access to rapid testing in underserved populations [2,24,58].

### 3.1. RNA and DNA Sequencing

DNA and RNA sequencing are crucial tools in infectious disease detection, particularly for point-of-care (PoC) diagnostics, where rapid and accurate results are essential. Various CMOS-based approaches have emerged to meet these demands, offering a range of technologies from label-free detection to high-throughput screening and single-molecule analysis.

#### 3.1.1. Electrochemical Sensing

Electrochemical sensors are widely used in disease detection applications due to their sensitivity and versatility. In this section, we will discuss several examples of these systems and their specific applications.

One significant advancement is the development of a wireless ultra-wideband system-on-chip (SoC) for DNA analysis, as described in [59]. This system eliminates the need for time-consuming PCR amplification by employing a label-free DNA detection mechanism. By integrating nanostructured electrodes with electrochemical sensors and an on-chip analog-to-digital converter (ADC), the system ensures high sensitivity in detecting DNA binding events. The use of cyclic voltammetry allows for precise signal quantification, and the real-time transmission of data via an ultra-wideband transmitter makes the system particularly suitable for PoC diagnostics. This design combines scalability and cost-efficiency, leveraging CMOS technology for rapid and accessible DNA analysis. Building on the foundation of electrochemical sensing, a CMOS-based biochip described in [60] introduces a 32 × 32 three-electrode voltammetry pixel array. This system is designed for high-throughput screening, allowing for the parallel processing of multiple DNA samples, a critical advantage during large-scale screening scenarios such as pandemic responses. The integration of a high-density sensor array with custom voltammetry circuits enables rapid, precise detection across multiple samples. While similar in its CMOS foundation to the system in [59], this biochip offers enhanced scalability and throughput, making it more suited to situations requiring mass testing.

Meanwhile, the system in [61] presents a CMOS polar mode biosensor designed for electrochemical detection using polar modulation techniques. This sensor features in-pixel averaging for enhanced signal quality, enabling precise measurements even in noisy environments. Unlike the fluorescence and nanopore-based approaches, this biosensor offers a novel method of electrochemical detection, emphasizing power efficiency and scalability for PoC applications. The integration of signal processing with polar modulation allows for high-fidelity data acquisition, positioning this technology as a strong candidate for real-time, cost-effective diagnostics.

#### 3.1.2. Fluorescence-Based Sensing

Fluorescence-based systems provide an essential approach for the ultra-sensitive detection of DNA and RNA, particularly in low-concentration samples. One such system, as described in [62], leverages CMOS technology to integrate photodetectors and analog amplification circuits for capturing weak fluorescence signals emitted by labeled nucleic acids. The combination of high-resolution ADCs and ultra-wideband (UWB) communication facilitates real-time signal transmission, making this platform especially effective for detecting low-abundance nucleic acids, critical for early-stage disease detection. This fluorescence-based method complements the more throughput-focused electrochemical systems discussed in [59,60] by emphasizing sensitivity over processing capacity. Further advancing the field, the work presented in [63] uses water-soluble conjugated polymers to detect DNA hybridization. By employing Förster resonance energy transfer (FRET), this method amplifies fluorescence signals, allowing for highly sensitive detection. The system can be implemented both on-chip and off-chip, offering flexibility for point-of-care (PoC) applications. This design also integrates the use of polymers such as poly(thiophene) and poly(fluorene-co-phenylene), which undergo conformational changes upon interaction with DNA, directly affecting their optical properties. This unique mechanism enables precise detection of subtle changes in DNA, such as single-nucleotide polymorphisms (SNPs), highlighting the potential of polymer-based systems for high-sensitivity genetic analysis.

In addition to these advances, the study conducted by [64] focuses on fluorescence-based strategies for detecting and quantifying DNA damage. This method avoids the use of radiolabeled compounds and instead employs fluorescent probes for real-time detection of DNA damage, enhancing both safety and sensitivity. The integration of DNA damage detection further broadens the application of fluorescence-based systems, making them indispensable for both disease diagnosis and genetic research. The combination of fluorescence techniques and CMOS technology, as seen in these systems, provides a powerful tool for early-stage disease detection, particularly in clinical and PoC settings.

#### 3.1.3. Nanopore-Based Sensing

In addition to electrochemical and fluorescence-based systems, nanopore-based technologies offer unmatched molecular precision. The system presented in [65] utilizes nanopore DNA sequencing integrated with a patch-clamp system to measure ionic currents generated as DNA molecules pass through the nanopores. This approach focuses on single-molecule detection, offering a level of precision that high-throughput systems cannot match. While the nanopore system excels in sensitivity, its lower throughput may limit its use in large-scale screening, making it more suited to specialized clinical diagnostics where molecular-level analysis is required. Other nanopore uses are described [66,67,68]. Overall, these CMOS-based systems—ranging from the high-throughput capabilities of electrochemical sensors to the ultra-sensitive fluorescence detection and polar mode biosensing—illustrate the diverse technological approaches to DNA and RNA analysis. The continued innovation in CMOS integration enables scalable, cost-effective solutions for infectious disease detection, with each system offering unique advantages depending on the specific diagnostic needs.

#### 3.1.4. Other Methods

Optical sensing is another critical method used for detecting molecular interactions, as demonstrated in [69]. This system utilizes micro-ring resonators (MRRs) to detect shifts in refractive index caused by molecular interactions, such as those involving bovine serum albumin (BSA) and anti-BSA antibodies. Unlike intensity-based approaches, the dual-ring phase-based architecture employed in [69] significantly enhances the sensitivity and accuracy of detection. The integration of both electronic and photonic components into a single SoC eliminates the need for external optical systems, making it a compact and scalable solution, ideal for real-time, label-free diagnostics in point-of-care (PoC) settings. This capability allows for precise biomolecular detection while maintaining a cost-effective design. In contrast, Ref. [70] integrates multiple types of sensors on a single CMOS chip, combining potential recording, optical sensing, and four-point impedance measurements. This system offers multi-modal sensing, providing a more holistic cellular characterization through a combination of source-follower amplifiers and optimized readout circuits for each modality. The four-point impedance sensing, in particular, enhances the accuracy of cell adhesion and morphology measurements, surpassing traditional methods. The system’s data conversion is handled by 10-bit SAR ADCs, with data transmitted via an SPI interface. This high level of integration, allowing the simultaneous processing of up to 1568 pixels in parallel, makes it well-suited for high-throughput applications such as drug testing. Both systems underscore the versatility and potential of integrated sensor platforms in advancing PoC diagnostics. While Ref. [69] focuses on optical detection for molecular sensing, Ref. [70] demonstrates the integration of various sensing methods on a single chip, allowing for broader applications in cellular analysis and high-throughput screening.

### 3.2. Antigen/Antibody Detection

Ref. [71] is a great example for antibody detection at PoC. It can detect multiple antibodies, such as those for rubella and mumps, in human serum using a high-density electrochemical biosensor array. The sensor employs a coulostatic discharge technique with interdigitated microelectrodes (IDEs) to amplify redox signals. The analog circuit design includes a low-leakage switch and a unity-gain buffer, which are crucial for measuring the slow discharge of the sensor’s double-layer capacitance. The system does not use a traditional ADC; instead, it relies on the slow voltage change during discharge as the signal. The data telemetry is simplified due to the unique discharge method, which allows for dense sensor arrays without complex external circuitry. In [72], the focus shifts towards antigen and antibody detection using a Lab-on-CMOS platform for ion channel characterization via nanopore arrays. This research is motivated by the need for high-throughput proteomics and biosensing applications. The system’s electrochemical interface circuit (EIC) is notable for its low-noise performance, detecting currents as low as 10 pA, which is critical for nanopore-based biosensing applications. Compared to the DNA sequencing works in the previous subsection, this design emphasizes low-power consumption and compact size while maintaining high sensitivity for ion channel measurements. It achieves this by placing the EIC directly beneath the nanopore chamber, thus reducing interface capacitance and improving signal-to-noise ratios. This system provides a significant advantage in high-throughput detection compared to the lower-density approaches used in DNA sequencing studies such as [59,73].

### 3.3. Pathogen Detection

Some devices are specifically designed for respiratory pathogens, including SARS-CoV-2 [62,73].

Ref. [73] introduces a Multiplex PCR CMOS biochip, specifically designed for detecting multiple pathogens, including SARS-CoV-2. While PCR amplification is used in this design, the motivation is to provide a highly scalable, portable solution capable of detecting multiple RNA or DNA sequences in a single reaction. The biochip integrates temperature control circuits for thermal cycling, amplifiers, ADCs for signal processing, and SPI telemetry for data communication. This paper compares favorably to [59,60] by addressing the simultaneous detection of multiple pathogens, whereas the previous works focus on single-target DNA analysis. However, its reliance on PCR, while enhancing sensitivity, adds complexity compared to label-free detection methods. The work in [71] describes a high-density biosensor array designed for pathogen detection using redox amplification and coulostatic discharge. This system stands out due to its innovative use of interdigitated electrodes (IDEs) for antigen-antibody interactions, enhancing the sensitivity of pathogen detection through redox activity. Compared to the DNA/RNA detection systems discussed in the earlier subsections, which rely on PCR or electrochemical detection, this approach leverages redox reactions to amplify weak molecular signals, providing higher sensitivity and faster results. Furthermore, its high-density array format enables parallel sensing of multiple analytes, offering scalability similar to the high-throughput system in [60]. However, Ref. [71] specifically targets the detection of antigens and antibodies, rather than nucleic acids, making it a complementary solution for applications where protein detection is prioritized over genetic material analysis.

### 3.4. Interface Design

**Figure 6:**
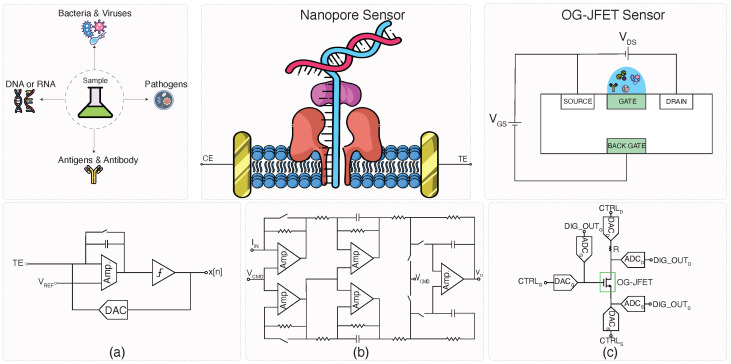
(**a**) ADC-direct interface of the fluorescence biochip for DNA and RNA testing [62]. (**b**) A patch-clamp ASIC interface for nanopore-based DNA analysis [65]. (**c**) OG-JFET interface for biochemical sensing [74].

Efficient sensor interface design is crucial for maximizing the performance of point-of-care (PoC) devices aimed at infectious disease detection. Figure 6 illustrates three key interface designs utilized for different biosensors, highlighting the importance of integrating signal amplification, data conversion, and control mechanisms within the system. Each of these interface designs is tailored for specific biosensor applications, but they also provide general principles that can be applied across various types of sensors.

#### 3.4.1. ADC-Direct Interface for Fluorescence-Based Biosensors

The circuit in panel (a) shows an analog-to-digital converter (ADC)-direct interface designed for fluorescence-based biosensors. In this design, weak fluorescence signals from DNA or RNA molecules are amplified and processed using an operational amplifier before being converted to a digital signal through a high-resolution ADC. The system utilizes a digital-to-analog converter (DAC) to control the voltage applied to the fluorescence detector, ensuring stable and accurate readings [62]. Although this interface is specifically designed for fluorescence-based sensors, the architecture can be generalized to other types of optical biosensors where weak signal amplification is necessary before digital processing. This system’s use of a direct ADC interface ensures high precision in detecting low-concentration analytes, making it ideal for early-stage disease detection.

#### 3.4.2. Patch-Clamp ASIC Interface for Nanopore Sensors

Panel (b) illustrates a patch-clamp ASIC interface tailored for nanopore-based DNA detection systems. This design employs an array of operational amplifiers and current mirrors to maintain stable voltage levels across the nanopore, allowing for the real-time measurement of ionic currents generated by DNA molecules as they pass through the nanopore [65]. The integration of a command voltage ($V_{C}MD$) and the feedback loop enhances the system’s stability, which is critical for achieving the high sensitivity required for single-molecule detection. While this specific implementation is used for nanopore sensors, the patch-clamp interface design can be extended to any biosensor where precise control of ionic current and feedback regulation is crucial for accurate signal measurement.

#### 3.4.3. OG-JFET Interface for Biochemical Sensing

Panel (c) shows an open-gate junction field-effect transistor (OG-JFET) interface used for biochemical sensing applications. In this configuration, the OG-JFET sensor is connected to a source and drain, with the gate exposed to the biochemical analyte of interest. The system leverages a combination of DACs, ADCs, and control signals to monitor the changes in current that occur as biochemical reactions take place near the sensor’s gate. This interface is designed to measure the redox current generated during electrochemical interactions, making it suitable for monitoring biochemical markers such as proteins or pathogens [53,63]. While the OG-JFET architecture is demonstrated here for a specific biochemical sensor, its modular design can be adapted to other electrochemical sensors that rely on real-time monitoring of current variations.

Each of these interface designs showcases how different biosensor technologies can benefit from tailored interface circuits that handle specific challenges, such as weak signal amplification, current measurement, or voltage regulation. Whether for optical, nanopore, or electrochemical sensors, integrating these interfaces with CMOS technology ensures that these systems can be miniaturized and optimized for portable, low-cost PoC devices. These versatile designs demonstrate their applicability across various infectious disease detection platforms, from DNA analysis to biochemical sensing. Table 2 summarizes some of the state-of-the-art designs for infectious disease detection applications.

## 4. Neural Interface PoC

Neural signals are the fundamental means of communication within the brain, enabling neurons to transmit information through electrical impulses. These signals, known as action potentials and local field potentials (LFPs), are generated by the movement of ions, such as sodium (Na+) and potassium (K+), across the neuron’s membrane. When a neuron is stimulated, sodium ions rush into the cell, causing depolarization, followed by the exit of potassium ions, which repolarizes the membrane and restores the neuron’s resting state. This process allows the neuron to generate an action potential, a rapid electrical signal that travels along the axon to communicate with other neurons. LFPs, on the other hand, represent the collective electrical activity of many neurons within a specific brain region, providing insights into the broader neural environment [75,76]. Understanding and harnessing these neural signals is crucial for detecting and treating neurodegenerative disorders, such as Alzheimer’s, Parkinson’s, and Huntington’s diseases, which affect over a billion people worldwide. These conditions lead to significant mortality and disability, making the development of innovative therapeutic strategies essential [77].

**Figure 7:**
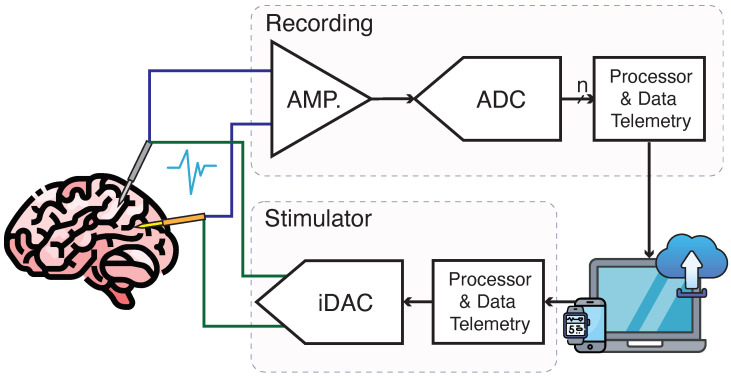
Block diagram of conventional neural interface microsystems.

Early generations of brain stimulation devices used an open-loop architecture, delivering electrical stimulation without accounting for the brain’s real-time state. This limitation restricted their effectiveness, as they could not adjust to the brain’s changing conditions [78,79]. However, recent advancements have introduced closed-loop systems that continuously monitor neural activity, allowing for more responsive and personalized treatment approaches [80,81]. An overview of neural recoring and stimulation is shown in Figure 7.

When designing neural recording systems, several key parameters must be considered to ensure effective performance. These include minimizing power dissipation to prevent tissue damage, achieving low noise levels for accurate signal amplification, and managing large DC offsets at the electrode-tissue interface. Additionally, amplifiers must capture a wide range of signal frequencies, from low-frequency LFPs to high-frequency action potentials, while being compact enough to fit within the limited silicon area for large-scale electrode arrays [82]. For simultaneous neural recording and stimulation, another important parameter has to be considered, which is the dynamic range (DR). Neural signals are up to a few millivolts. However, if we want to enable recording while stimulating, artifacts as large as 200 mV can be sensed at the recording electrode. In order to prevent saturation in the presence of these artifacts, high DR channels should be designed [83,84]. In this section, we discuss some examples of neural interfaces that have been developed to interact with the central nervous system. These examples highlight the various ways in which these technologies are being used to better understand brain function, treat neurological disorders, and improve patient outcomes. By exploring these applications, we can gain a deeper insight into the potential and challenges of neural interfaces in modern medicine. Table 3 shows a summary of state-of-the-art neural interface ICs which are developed in CMOS.

### 4.1. High Dynamic Range Neural Recording

**Figure 8:**
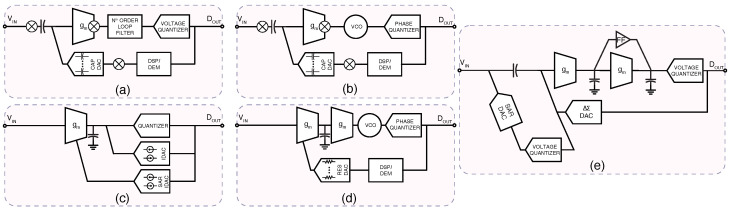
(**a**) Second-order ΔΣ ADC [85]. (**b**) VCO-based ΔΣ ADC [86]. (**c**) SAR-assisted ΔΣ ADC [87]. (**d**) SNDR Gm-C-based ΔΣ modulator with a feedback-assisted Gm linearization [88]. (**e**) Dynamic-zoom ΔΣ ADC [89].

Examples of high dynamic range neural recording channels include several innovative approaches that leverage advanced ADC techniques to enhance signal quality and resolution. Chandrakumar et al. [84] employed a conventional third-order ΔΣ ADC and a low-noise amplifier, achieving a DR greater than 80 dB. However, the power consumption was not as low as required for the high number of channels used. Therefore, an ADC-direct approach was proposed in which the amplifier stage is removed and the electrode connects to the converter directly. The block diagram of this work is shown in Figure 8a [85]. The design utilized a direct-ADC method, incorporating auto-ranging to improve the signal-to-noise ratio (SNR) of a second-order ΔΣ ADC. In this design, the DAC within the ΔΣ ADC dynamically adjusts its resolution based on incoming data, allowing for faster artifact recovery while maintaining high SNR and capturing neural signals with high resolution.

Ref. [86] shown in Figure 8b used a VCO-based approach. VCO-based ΔΣ ADCs are proposed to enable the possibility of higher DR as technology advances and supply becomes smaller. However, in this approach, linearity becomes a significantly more challenging issue. Ref. [87], in Figure 8c, also follows a direct ADC approach, utilizing a hybrid ADC where a SAR ADC facilitates rapid artifact recovery in tens of microseconds, while a fine ΔΣ stage achieves approximately 50-dB resolution in neural signal digitization.

Ref. [88], shown in Figure 8d, is another attempt at VCO-based ADCs, but they have used Gm-C as the first integrator and VCO as the second integrator to enhance the resolution. Further innovations include a fourth-order ΔΣ modulator developed by [89] illustrated in Figure 8e, which integrates a two-step summation technique and dual cycle shift (DCS) data weighted averaging (DWA) to enhance performance.

### 4.2. Neural Interface Microsystems

This section reviews several neural interface ICs that extend beyond basic stimulation or recording channels, developing microsystems suitable for PoC applications. A notable bidirectional neural interface circuit supports both recording and stimulation while minimizing artifacts and common-mode noise. It features a common average referencing (CAR) front-end for noise suppression and a range-adapting (RA) SAR ADC for efficient data conversion. Active stimulation artifact cancellation ensures signal integrity during simultaneous recording and stimulation, with a low-noise amplification of neural signals. The system also features dynamic power optimization through the RA SAR ADC and efficient telemetry for high signal-to-noise ratio (SNR) neural acquisition, suitable for long-term implantable use.

**Figure 9:**
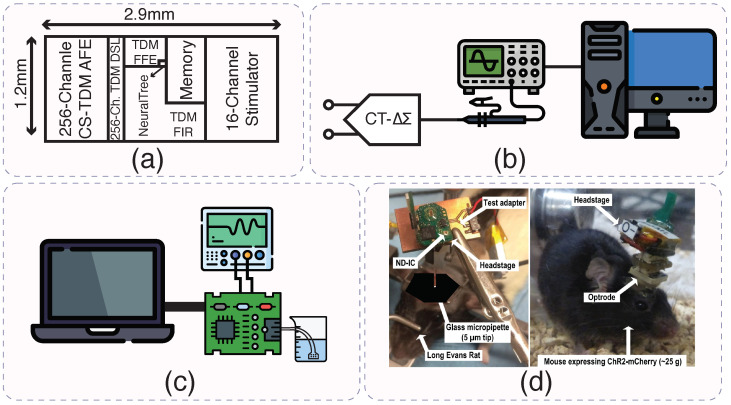
(**a**) Overview of closed-loop neuromodulation SoC of [90]. (**b**) High dynamic range neural recording chip experimental setup on a mouse illustration of [88]. (**c**) Time-based neural-recording IC with degeneration test setup illustration [54]. (**d**) Wireless electro-optic headstage in vivo setup [94].

Ref. [90] presents a 256-channel system-on-chip (SoC) designed for brain activity classification and closed-loop neuromodulation. The system integrates several innovative features, including a dynamically addressable analog front-end (AFE) that records neural signals, a NeuralTree classifier for accurate classification, and a high-voltage compliant neurostimulator for therapeutic intervention. The design emphasizes energy efficiency, scalability, and versatility, allowing for high-density neural recording and real-time classification with reduced hardware complexity. The SoC demonstrates significant advancements over previous systems in terms of channel count, power efficiency, and flexibility in handling various neural classification tasks. The chip photograph is shown in Figure 9a.

Shin et al. developed a 256-channel SoC capable of brain activity classification and closed-loop neuromodulation [91]. This system integrates a chopper-stabilized time-division-multiplexed (CS-TDM) front-end with delta-sigma (ΔΣ) modulation, achieving a low power consumption of 387 µW, while providing high-density recording for detailed neural signal acquisition. The system’s real-time classification and adaptability are crucial for applications in treating neurological disorders where precision is key.

Comparatively, Mendrela et al. introduced a bidirectional neural interface circuit that supports simultaneous neural recording and stimulation, while offering advanced artifact cancellation and noise suppression [92]. Their system, employing a range-adapting SAR ADC and a common average referencing (CAR) front-end, improves signal-to-noise ratio (SNR) by 39.8 dB, with ultra-low power consumption of just 330 nW per channel, making it highly efficient for neuromodulatory applications where minimizing interference is critical.

Lee et al. addressed the challenges of electrode offsets and artifacts in their continuous-time delta-sigma modulator (CTDSM), which employs Gm-C-based amplifiers for artifact-tolerant neural recordings [88]. Their system, consuming 6.5 µW, achieves robust performance with an SNDR of 80.4 dB over a 10 kHz bandwidth, making it ideal for environments with high artifact presence. They have tested their IC on a mouse for verification and the setup is shown in Figure 9b.

Similarly, Jeon et al.’s design focuses on maintaining high-fidelity recordings with a degeneration R-DAC and time-based quantization method to suppress stimulation artifacts and electrode offsets [54]. Their system achieves a peak SNDR of 81.3 dB over a 200 Hz bandwidth, while consuming 3.9 µW per channel, making it particularly suitable for implantable devices that prioritize both precision and low power consumption. The experimental setup of this IC is shown in Figure 9c.

Wireless capabilities are a crucial development in this field. The presence of a physical connection between electrodes and monitoring equipment limits the mobility of the subject under study and necessitates transcutaneous wires that present a risk of infection [93]. Gagnon-Turcotte et al. developed a wireless electro-optic headstage for closed-loop optogenetics [94]. This electro-optic headstage is tested on a mouse, which is shown in Figure 9d. By integrating a digital wavelet transform (DWT) neural signal decoder with real-time classification, this system allows for wireless, artifact-free monitoring in freely moving animal models, advancing both neurological research and clinical applications.

Seizure detection accuracy and timeliness are critical for smart healthcare. Traditional methods often rely on cloud-based data processing, which can introduce latency and potential privacy concerns. The eSeiz device in [95], developed as an edge-computing solution, addresses these challenges by performing real-time seizure detection directly on the device, reducing the need for constant communication with external servers. By employing lightweight machine-learning algorithms, this system enables on-device processing while maintaining high detection accuracy, as tested on clinical datasets. The edge-device architecture enhances privacy, reduces latency, and is ideal for deployment in both home and clinical environments.

Finally, systems like Kim et al.’s sub-μW/channel analog front-end, which utilizes a spike-driven data compression technique, highlights the importance of power efficiency for implantable devices [96]. Their system achieves an impressive 89% data compression while consuming only 0.88 µW/channel, offering a highly efficient solution for long-term neural recording applications.

In conclusion, these neural interfaces show significant progress in addressing power consumption, artifact cancellation, and wireless data transmission. From closed-loop neuromodulation to wireless real-time recording, these innovations are transforming PoC neural monitoring systems.

**Table 3:** Comparison of selected technologies used in neural interfaces.

**Ref.**	[92]	[54]	[88]	[94]	[95]	[96]	[97]
**Applications**	Brain activity classification and closed-loop neuromodulation	Bidirectional neural interface	Simultaneous neural recording and electrical stimulation	Artifact-tolerant neural recording interfaces	Wireless electro-optic headstage for closed-loop optogenetics	Seizure detection system	Implantable neural recording IC
**Targets**	Neural signals in general	Electrocorticography (ECoG) and LFP signals	LFP	Neural signals, including local field potentials (LFPs)	Neural recording (APs) and real-time optogenetic stimulation	LFP	Neural signals including spikes and APs
**Tech.**	65 nm	180 nm	180 nm	65 nm	130 nm	NR	180 nm
**Input Range [mV]**	±50	NR	200	300	NR	NR	NR
**IRN [µVrms]**	NR	3.05	91.9	95	NR	NR	5.4
**Bandwidth [Hz]**	NR	2k	200	10k	NR	3–29	6.4
**Total DR [dB]**	NR	NR	91.2	80.4	NR	NR	48
**Stimulation**	On-Chip	Yes	On-Chip	No	On-Chip	No	No
**Processor**	On-Chip	Yes	No	No	On-Chip	On-Chip	On-Chip
**Resolution**	NR	NR	NR	NR	16-bit	NR	48 dB
**Power [µW]**	1.51	0.33	3.9	6.5	56.9	39.5	0.88
**Area [mm^2^]**	0.014	0.17	0.225	0.078	0.08	NR	0.018

## 5. Commercialized PoC ICs and Devices

### 5.1. Commercialized ICs

Biomedical integrated circuits (ICs) have revolutionized the development of point-of-care (PoC) devices, enabling real-time, low-cost monitoring solutions. The front-end design of these systems is often complex, requiring precise handling of signals, data acquisition, and communication. To address this challenge, companies like Analog Devices and Thomson have introduced front-end circuits specifically designed for biomedical applications. Their ICs, such as the AD5940 and AD5941, have become integral to PoC devices, offering robust signal conditioning and data acquisition capabilities. These ICs are widely adopted due to their ability to perform complex electrochemical and impedance measurements, crucial for a wide range of medical applications.

For example, the AD5940 has been utilized in continuous bioimpedance monitoring systems, improving accuracy through calibration techniques, and in a portable system for detecting A549 lung cancer cells, where its electrochemical measurement capabilities were key to the success of the design [98,99]. Similarly, the SenSARS device, designed for detecting SARS-CoV-2, leverages the high impedance-handling capabilities of the AD5940 to deliver accurate, real-time diagnoses in a portable form factor [100]. These ICs have proven to be effective building blocks for many PoC applications, reducing development time and allowing for highly precise measurements, all while remaining cost-effective.

### 5.2. Commercialized Devices

**Figure 10:**
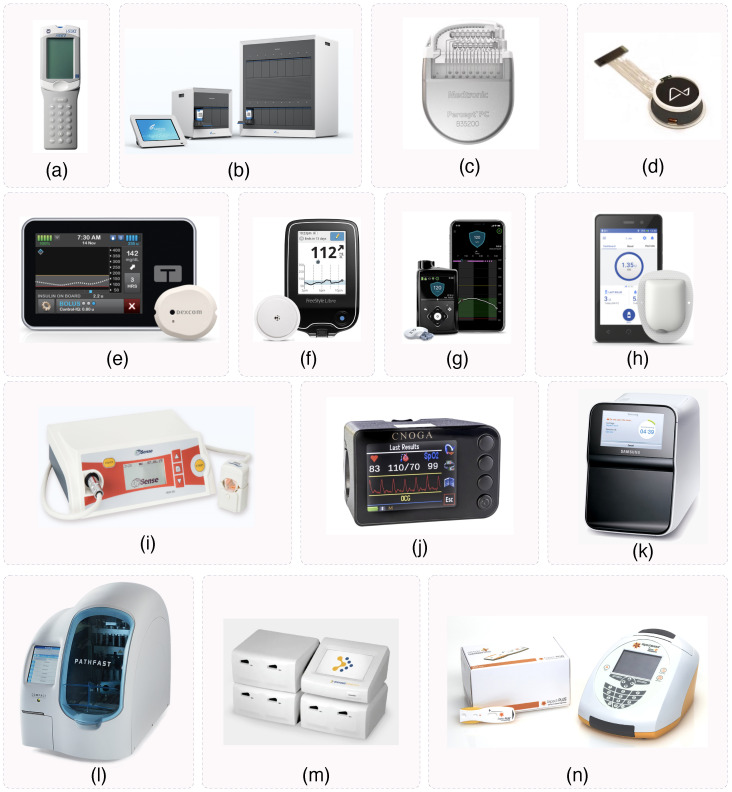
Commercialized PoC devices: (**a**) i-STAT Abbot [108]; (**b**) Cepheid’s GeneXpert [109]; (**c**) Medtronic’s Percept PC Neurostimulation [110]; (**d**) Neuralink Brain Implant [111]; (**e**) Tendem Diabetes Care T:Slim X2 Insuling Pump [112]; (**f**) Abbot FreeStyle Libre [113]; (**g**) Medtronics MiniMed 770G [114]; (**h**) Omnipod DASH Insulin Management System [115]; (**i**) OrSense NBM 200 [103]; (**j**) CNOGA MTX [102]; (**k**) Samsung LABGEO PT10 [104]; (**l**) Pathfast [105]; (**m**) RAMP system [106]; (**n**) Aspect Plus—ST2 [107].

Another level of commercialization involves fully integrated PoC devices, where not only the front-end ICs are standardized, but the entire system is designed for accessibility and ease of use. These commercialized devices are shown in Figure 10. For instance, the i-STAT by Abbott is a handheld PoC device widely used for rapid blood analysis, offering near-instant results for parameters such as electrolytes and blood gases. This system has become a staple in hospitals, ambulances, and remote care settings due to its portability and speed [101]. Similarly, Cepheid’s GeneXpert System, which uses PCR testing, is highly effective for detecting infectious diseases such as SARS-CoV-2, tuberculosis, and HIV. Its compact design and disposable cartridges make it ideal for both hospital and point-of-care settings, providing results in less than an hour [101].

In the field of glucose monitoring, the Medtronic MiniMed 770G and Abbott FreeStyle Libre systems have set the standard for managing diabetes through continuous glucose monitoring (CGM) combined with automated insulin delivery. These devices not only track glucose levels in real time but also integrate seamlessly with smartphones and wearables, enhancing convenience for patients. The Omnipod DASH Insulin Management System also contributes to this trend by offering a tubeless, discreet solution for insulin delivery [101]. Additionally, Cnoga Medical’s MTX Monitoring Device provides non-invasive blood monitoring solutions for various physiological parameters, representing a new era of PoC diagnostics [102]. OrSense products further enhance the landscape of non-invasive monitoring with their hemoglobin and oxygen saturation measurement technologies, demonstrating another innovative approach to patient-friendly healthcare solutions [103]. Samsung LABGEO PT10 is a notable example of a commercialized PoC device designed for clinical use, offering a compact, portable solution for blood chemistry analysis [104]. Pathfast’s immunoanalyzer also exemplifies an innovative approach to PoC testing by enabling rapid, reliable results for a wide range of diagnostic tests [105]. Moreover, Response Biomedical’s systems offer rapid on-site diagnostics with their easy-to-use, portable systems for detecting cardiac markers and infectious diseases [106]. Finally, Sopachem’s Aspect Plus ST2 Rapid Test Reader introduces a new approach to rapid, point-of-care diagnostic tools with its fast, accurate test results in clinical settings [107]. These commercialized devices are prime examples of how PoC technologies are becoming more integrated into everyday healthcare, providing life-saving treatments with minimal intervention.

Moreover, the development of neural interfaces is advancing rapidly. Neuralink’s work on brain-computer interfaces aims to monitor brain activity and eventually control external devices wirelessly. Medtronic’s Percept PC Neurostimulator, already in commercial use, offers deep brain stimulation for patients with neurological conditions, using brain-sensing technology to tailor therapy to individual needs [101]. Such devices illustrate the growing commercial presence of neural interfaces in healthcare, bridging the gap between research innovations and patient treatments.

As these examples show, the commercial adoption of PoC devices is on the rise. From front-end ICs to fully integrated devices, the advancement of CMOS technology is making these devices more affordable, accessible, and reliable. The success of biomedical ICs like the AD5940 in enabling precise, real-time data acquisition, combined with the broader commercialization of complete systems, represents a significant leap forward in healthcare innovation. Future development efforts should focus on improving integration between sensors, data telemetry, and power management systems to fully realize the potential of these devices in clinical and home settings.

## 6. Future Directions

Future advancements in point-of-care (PoC) devices will focus on several key areas to enhance performance and broaden their applications. One major direction is the integration of sensor circuits and systems, allowing for fully autonomous operation by combining sensing, signal amplification, data telemetry, and communication within a single compact platform. These integrated systems will enable real-time diagnostics and seamless communication with external devices, paving the way for more efficient, wireless healthcare solutions [116,117].

Lowering the power consumption and reducing the physical footprint of PoC devices will also be critical. Innovations in ultra-low-power circuits and advanced energy management will extend the battery life of wearable and implantable devices, making continuous, long-term monitoring more feasible. At the same time, reducing the size of these devices through efficient use of silicon and advanced packaging technologies will allow for more portable and discreet designs, further expanding their applications in wearable healthcare [118].

In the coming years, the scope of PoC devices will also expand as new diagnostic applications are discovered. Beyond medical diagnostics, these technologies are expected to play a growing role in personalized medicine, environmental monitoring, and even veterinary care, offering rapid, on-site diagnostic capabilities in various fields [119,120,121].

Additionally, the combination of novel microfluidic systems and microelectronics will play a critical role in PoC evolution. Microfluidics will enable precise fluid handling in minimal volumes, while advancements in microelectronics will drive the development of highly sensitive, low-power detection mechanisms. This synergy will allow PoC devices to achieve unprecedented levels of accuracy and efficiency [122,123].

Furthermore, the integration of Internet of Things (IoT) technologies and artificial intelligence (AI) will revolutionize PoC devices. IoT connectivity will allow these devices to collect and transmit data wirelessly in real time, while AI algorithms can process and analyze the data, enabling predictive diagnostics, personalized treatment plans, and remote patient monitoring. The combination of IoT and AI will transform PoC devices from standalone diagnostic tools into fully connected, intelligent systems that support continuous, real-time health monitoring and advanced decision making [124,125,126].

## 7. Conclusions

This review underscores the pivotal role that point-of-care (PoC) devices play in enhancing the accessibility and quality of healthcare, especially in resource-limited environments. With ongoing advancements in CMOS technology, the integration of sensors, front-end circuits, and back-end processing units has resulted in the development of more compact, cost-effective, and power-efficient PoC systems. These innovations are transforming healthcare by enabling real-time diagnostics and continuous health monitoring for a wide range of conditions, from chronic diseases like diabetes and neurological disorders to the early detection of infectious diseases.

In this review, we provide a comprehensive overview of PoC devices and their diverse applications. We begin by discussing the detection of various biomarkers, followed by an exploration of sensor technologies, analog interfaces, and communication methods that facilitate data acquisition and processing in these systems.

Additionally, we highlight several commercial PoC devices, illustrating how cutting-edge research is transitioning from the laboratory to clinical practice, revolutionizing healthcare delivery. As research and technological advancements continue, the capabilities of PoC devices will expand, further enabling personalized, accessible, and efficient healthcare solutions for a broader population.

## Figures and Tables

**Table 1:** Comparison of selected technologies used in blood analysis.

Ref.	[41]		[40]	[33]	[47]	[37]	[46]	[48]	[52]	[34]
Applications	Personalized Pharmacokinetics			Neuroscience				In vivo Monitoring		Pathobiology of diseases.
Targets	Small-molecule drugs			Dopamine				Glucose/Ethanol/H2O2		Dopamine and Uric Acid
Recognition element	Structure-switching aptamers			None (limited to electroactive molecules)		ISFET	Graphene	Enzyme (GOx/Aox)		Molecules
Tech.	65 nm		65 nm	65 nm	65 nm	0.35 µm		65 nm	65 nm	
Read-out method	SWV (Square-Wave Voltammetry)	CA (Chronoamperometry)	SWV	FSCV (Fast Scan Cyclic Voltammetry)	FSCV	Self-oscillating circuit	CV (Cyclic Voltammetry)	Amp.	CA	Voltammetry
Electrode, Area	Au, 0.25 mm^2^		Au, 0.25 mm^2^	Graphene, 1000 µm^2^	CFM, 2400 µm^2^	Ag/AgCl, 10 µm^2^	Graphene as WE Ag/AgCl as RE	Pt, 0.0144 mm^2^	Au, 0.025 mm^2^	GP5AuNPs5, 0.051 cm^2^
Potentiostat	On-chip		On-chip	On-chip	n.a.	n.a.	Integrated	On-chip	On-chip	n.a.
Waveform Generator	On-chip		On-chip	Off-chip	Off-chip	On-chip	Integrated	On-chip	On-chip	n.a.
Sensor IRN (SNR=1)	4.36 nArms	15.2 pArms	1.6 nArms	20 pArms	92 pArms	n.a.	n.a.	100 pArms	1.24 nArms	n.a.
Bandwidth	2.5 kHz	2.5 kHz	2 kHz	1 kHz	2 kHz	n.a.	n.a.	n.a.	n.a.	n.a.
Imax	±800 nA	±2.5 nA	±800 nA	±2.56 nA	±430 nA	6 µA	n.a.	350 nA	80 nA	n.a.
Total DR (Imax/Inoise, rms)	100 dB		60 dB	108 dB	79.4 dB	43 dB	n.a.	70.9 dB	36.1 dB	n.a.
Electrochemical Data Acquisition Rate	0.5 Hz	5 Hz	0.5 Hz	100 Hz	100 Hz	n.a.	n.a.	n.a.	n.a.	n.a.
Power	5.25 mW	0.22 mW	6.64 mW	36 µW	14.4 µW	n.a.	n.a.	4 µW	0.97 µW	n.a.

**Table 2:** Comparison of selected technologies used in infectious disease detection.

**Ref.**	[62]	[73]	[61]	[69]	[71]
**Applications**	Medical Diagnostics	Detection of Upper Respiratory Pathogens	Detection of Ebola, Zika, etc.	PoC for Fast-Spreading Diseases	Infectious Disease Detection (Vaccine Screening)
**Targets**	Flu, RSV, HPIV, …	DNA/RNA of Upper Respiratory Pathogens (COVID, FluA, FluB, RSV)	DNA Hybridization for Detecting the Zika Virus	Pathogens	Antibody
**Recognition Element**	Fluorescence Biosensing Pixel Array	Biosensing Pixels	Electrochemical CMOS	Microring Resonators (MRRs)	Electrochemical Sensor
**Tech.**	0.25 µm CMOS	CMOS	180 nm	45 nm RFSOI	TSMC 180 nm
**Read-out Method**	Continuous Wave (CW) Fluorescence-Based Detection	Quantitative Polymerase Chain Reaction (PCR) Technique	Measurement of Impedance Change Between Electrode and Solution	Resonant Shift by Molecular Binding	Redox-Amplified Coulostatic Discharge
**Sensor Input Noise**	5.5 pA @ 90 Hz	NR	NR	170 pA	NR
**Bandwidth (Hz)**	50	NR	5k–1M	25 MHz (assuming Nyquist rate)	5k–1M
**Imax**	NR	10 nA	NR	100 µA	NR
**Total DR (Imax/Inoise, rms)**	116 dB	137 dB	NR	100 µA/10 nA	40 dB
**Data Communication**	NR	SPI	SPI	No	NR
**Power**	118 mW	25 mW	197 µW	NR	63 µW
